# Feline low-grade alimentary lymphoma: an emerging entity and a potential animal model for human disease

**DOI:** 10.1186/s12917-018-1635-5

**Published:** 2018-10-11

**Authors:** Mathieu V Paulin, Lucile Couronné, Jérémy Beguin, Sophie Le Poder, Maxence Delverdier, Marie-Odile Semin, Julie Bruneau, Nadine Cerf-Bensussan, Georgia Malamut, Christophe Cellier, Ghita Benchekroun, Laurent Tiret, Alexander J German, Olivier Hermine, Valérie Freiche

**Affiliations:** 1 Université Paris-Est, École Nationale Vétérinaire d’Alfort, 7 Avenue du Général de Gaulle, 94700 Maisons-Alfort, France; 2 Hematology Department, Hôpital Universitaire Necker – Enfants Malades, Assistance Publique – Hôpitaux de Paris (APHP), Paris, France; 3 Université Paris Descartes, Sorbonne Paris Cité, Paris, France; 4 INSERM UMR 1163, CNRS ERL 8254, Institut Imagine, Paris, France; 5 Internal Medicine Department, Université Paris-Est, École Nationale Vétérinaire d’Alfort, 7 Avenue du Général de Gaulle, 94700 Maisons-Alfort, France; 6 UMR 1161 Virologie, INRA-ENVA-ANSES, Université Paris-Est, École Nationale Vétérinaire d’Alfort, Maisons-Alfort, France; 7 Anatomical Pathology Department, Université de Toulouse, École Nationale Vétérinaire de Toulouse, 23 Chemin des Capelles, 31076 Toulouse Cedex, France; 8 Pathology Department, Hôpital Universitaire Necker – Enfants Malades, Assistance Publique – Hôpitaux de Paris (APHP), Université Paris Descartes, Sorbonne Paris Cité, Paris, France; 9 INSERM 1163, Institut Imagine, Site Hôpital Universitaire Necker – Enfants Malades, Paris, France; 10 UMR 1163, Laboratory of Intestinal Immunity, INSERM, Paris, France; 11 Gastroenterology Department, Hôpital Européen Georges Pompidou, Assistance Publique – Hôpitaux de Paris (APHP), Université Paris Descartes, Sorbonne Paris Cité, Paris, France; 12 INSERM UMR 1163, Institut Imagine, Paris, France; 13 Inserm U955-E10 BNMS, IMRB, Université Paris-Est, École Nationale Vétérinaire d’Alfort, 94000 Maisons-Alfort, France; 14 Institute of Ageing and Chronic Disease, University of Liverpool, Leahurst Campus, Chester High Road, Neston, CH64 7TE UK

**Keywords:** Comparative oncology, Cat, Inflammatory bowel disease, Human indolent digestive T-cell lymphoproliferative disorder

## Abstract

**Background:**

Low-grade alimentary lymphoma (LGAL) is characterised by the infiltration of neoplastic T-lymphocytes, typically in the small intestine. The incidence of LGAL has increased over the last ten years and it is now the most frequent digestive neoplasia in cats and comprises 60 to 75% of gastrointestinal lymphoma cases. Given that LGAL shares common clinical, paraclinical and ultrasonographic features with inflammatory bowel diseases, establishing a diagnosis is challenging. A review was designed to summarise current knowledge of the pathogenesis, diagnosis, prognosis and treatment of feline LGAL. Electronic searches of PubMed and Science Direct were carried out without date or language restrictions.

**Results:**

A total of 176 peer-reviewed documents were identified and most of which were published in the last twenty years. 130 studies were found from the veterinary literature and 46 from the human medicine literature. Heterogeneity of study designs and outcome measures made meta-analysis inappropriate. The pathophysiology of feline LGAL still needs to be elucidated, not least the putative roles of infectious agents, environmental factors as well as genetic events. The most common therapeutic strategy is combination treatment with prednisolone and chlorambucil, and prolonged remission can often be achieved. Developments in immunohistochemical analysis and clonality testing have improved the confidence of clinicians in obtaining a correct diagnosis between LGAL and IBD. The condition shares similarities with some diseases in humans, especially human indolent T-cell lymphoproliferative disorder of the gastrointestinal tract.

**Conclusions:**

The pathophysiology of feline LGAL still needs to be elucidated and prospective studies as well as standardisation of therapeutic strategies are needed. A combination of conventional histopathology and immunohistochemistry remains the current gold-standard test, but clinicians should be cautious about reclassifying cats previously diagnosed with IBD to lymphoma on the basis of clonality testing. Importantly, feline LGAL could be considered to be a potential animal model for indolent digestive T-cell lymphoproliferative disorder, a rare condition in human medicine.

## Background

Lymphoma is a clonal expansion of neoplastic lymphocytes in solid organs and is the most common feline neoplasm [1–7]. Feline lymphomas are usually classified according to anatomical location, with various types recognised including mediastinal, multicentric, and extra-nodal. A final type, alimentary lymphoma (AL) targets the gastrointestinal tract with variable involvement of extra-intestinal sites including lymph nodes, liver, spleen [3, 4, 7, 8]. Not only is this the most common anatomical form of lymphoma (50 to 75%), it is also the most common alimentary neoplasia in cats [1–5, 8–19].

Several subtypes of AL can be defined according to the histological grade (low, intermediate or high), cell size (small or large) and phenotype (T or B) of the neoplastic lymphocytes [6, 8, 14, 18, 20–23]. Originally, lymphomas were classified according to the World Health Organization (WHO) scheme into Enteropathy-Associated T-cell Lymphoma (EATL) type 1 and 2. Recent studies in human medicine indicate that EATL consists of two diseases that are morphologically and genetically distinct. They also differ in how frequently they are associated with coeliac disease [24]: EATL type I (80–90% of EATL) is strongly associated with coeliac disease, has usually a large-cell or pleomorphic cytology and may express CD30 [24]. EATL type II (10–20% of EATL) is less frequently associated with coeliac disease and is characterised by monomorphic population with frequent expression of CD56 [24]. EATL type II has been recently renamed as Monomorphic Epitheliotropic T-cell Lymphoma (MEITL), calling into question its relationship with classical EATL and implying that it might be best to consider it as a separate entity [25].

The current classification system for this disease has been defined by Moore et al. and distinguishes two entities [6]: first, “mucosal lymphomas” which are usually low-grade forms of alimentary lymphoma (LGAL) and are predominantly of small T-cell type (nuclear diameter < 2 red cell diameters) that Moore et al. found to match the WHO entity EATL type II. Second, “transmural lymphomas”, which are more frequently high-grade alimentary lymphomas (HGAL) and are composed of small or large cells that can be of B- or T-cell type. Transmural intermediate to large-sized T-cell lymphomas would match the WHO entity EATL type I for Moore et al. When of large T-cell type, these lymphomas are mostly Large Granular Lymphocytic Lymphomas (LGLL) and express the cytotoxic granule protein, granzyme B. LGLL is less frequent, but the most aggressive subform of AL and can be considered as a separate histological sub-classification of AL with LGAL and HGAL [8, 26, 27].

LGAL may be the most common subtype in cats, representing 60 to 75% of AL [18–20]. However, the real incidence is not accurate since it is based upon a few small sample studies. Moreover, for unknown reasons, it appears to be an emerging entity with an increasing incidence over the last ten years [8, 28]. The aim of this review is to provide an overview of current knowledge regarding the aetiology, clinical and biological presentation, diagnosis, treatment and prognosis of LGAL. Comparisons of this condition with the rare human disease, indolent T-cell lymphoproliferative disorder (LPD) of the gastrointestinal tract, will also be highlighted.

## Methods

Electronic searches of PubMed and Science Direct were carried out without date or language restrictions. A total of 176 peer-reviewed documents were identified and most of which were published in the last twenty years. 130 studies were found from the veterinary literature and 46 from the human medicine literature. Heterogeneity of study designs and outcome measures made meta-analysis inappropriate.

## Results

### Aetiopathogenesis

The aetiopathogenesis of feline LGAL is poorly understood. Several factors have been implicated as possible causes, but their involvement remains inconclusive, particularly considering the relationship with the AL histological grade and phenotype [14].

#### Feline retroviruses

An infectious aetiology has been suggested because the risk of developing AL is greater in cats infected by feline retroviruses (feline leukaemia virus [FeLV] or feline immunodeficiency virus [FIV]). However, this hypothesis has been challenged on account of the fact that most lymphocytic cancers in all species are non-retroviral-associated [29].

FeLV infects the lymphoid tissue, intestine and bone marrow [3], and is suspected to be a major risk factor in development of leukaemia and lymphoma in cats, particularly T-cell lymphoma with a mediastinal location [30, 31]. Immunohistochemical studies have identified that 50 to 70% of all feline lymphomas are positive for FeLV [3, 7, 13, 32, 33] and its presence is associated with poor prognosis in all lymphoma subtypes [2, 34]. The exact mechanisms by which FeLV causes neoplastic transformation are not known but could be related to virus genome insertion, resulting in modulation of neighbouring oncogenic or tumour suppressor gene expression [30, 35]. Further, in contrast to multicentric lymphoma, the exact role of FeLV in development of AL is also unclear since it is inconsistently identified in AL cases [34, 36, 37], and many forms of lymphoma, including AL, can effectively develop without exposure to FeLV in pathogen-free cats [2, 10]. Over the past 30 years, circulating FeLV antigen has been observed in 2 to 30% of cats with AL [3, 20, 36–38] but, more recently, most AL cases do not have circulating FeLV antigen [13]. One possible explanation for this is the success of widespread implementation of test and vaccination programmes, which have helped to decrease FeLV prevalence in cats [2, 5, 20, 39].

Using immunohistochemistry (IHC), only 3% of AL tumours are positive for FeLV [20, 37, 40] although, in one study involving 25 cats with AL, alimentary lymphoma provirus sequences were detected in approximately 1/3 of B-cell AL and almost 2/3 of T-cell AL [36]. These latter findings suggest that FeLV may be present in a latent or replication-defective form in some cases [36, 40]. In contrast, no provirus form of FeLV was detected in another study involving 32 AL cases, 30 of which were FeLV-antigen-negative [38]. The discrepancies between both studies could be due to variability in assay sensitivity, with the higher prevalence of FeLV provirus detection in the first study possibly result of use of a highly-sensitive semi-nested PCR assay [36]. Additional epidemiological, functional and highly-sensitive molecular analyses are therefore required to clarify the role that FeLV plays in AL. In particular, it would be helpful to clarify the association between the presence of FeLV and different histological grades of AL, especially LGAL given its increasing prevalence.

Like human (HIV) and simian (SIV) immunodeficiency viruses [41–43], FIV can induce indirect immune dysregulation, resulting in partial loss of antitumoral immunity and ultimately promoting tumour development [3, 44, 45]. Studies have shown that FIV infection may increase the risk of developing various types of lymphoma, including AL [7, 14, 34, 46]. In addition, components of the FIV genome were detected in tissues from 7 of 8 AL that had developed in FIV-positive cats [47], 4 and 3 of which were B- and T-lymphoma, respectively. Various parts of the gastrointestinal tract were affected, and there was mesenteric lymph node involvement in 3 cats. However, unfortunately histological grade was not determined.

A final virus that has been implicated as an aetiological agent is the panlymphotropic *Felis catus* gammaherpesvirus 1 (FcaGHV1), and this virus is known to infect at least one quarter of cats. A possible role is plausible given the detection of two human gammaherpesviruses (Epstein-Barr and Kaposi’s sarcoma-associated viruses) in some lymphoma cases [48, 49]. However, in one study, neither detection of FcaGHV1 DNA nor whole blood virus load was related to a diagnosis of lymphoma [50]. That said, the presence of circulating FcaGHV1 DNA was associated with significantly shorter survival compared with FcaGHV1 qPCR negative cases [50]. A limitation of this study was the fact that the criteria used to recruit lymphoma cases excluded lymphocytic low-grade gastrointestinal lymphoma and, as a consequence, further studies are required.

#### Bacterial infections

Over the last two decades, bacterial mucosal colonisation, particularly involving argyrophilic organisms such as *Helicobacter* spp., has been highlighted as a potential oncogenic factor in feline gastric cancer as a result of chronic antigenic stimulation [51, 52]*. Helicobacter pylori* infection is strongly associated with development of adenocarcinoma and mucosa-associated lymphoid tissue (MALT) lymphoma in humans [53, 54]. The gastric mucosa of dogs is often colonised by non-*Helicobacter pylori* helicobacters. Although their pathogenic significance in dogs is poorly understood, there is evidence of *Helicobacter spp*. infection in laboratory beagle dogs resulting in gastric lymphoid follicle formation that is considered a precursor of MALT lymphoma in humans [55].

Gastric *Helicobacter heilmannii* (Hhe) strains were reported to promote feline gastric lymphoma in one study involving 47 cats, where respectively 14 out of 16 and 2 out of 16 lymphoblastic and lymphocytic lymphomas were positive for Hhe organisms (especially the Hhe2 and Hhe 4 strains) [52]. Amongst a population of 33 low-grade T-cell epitheliotropic small intestinal lymphomas, mucosa-invasive bacteria and serosal colonisation have been identified in 18% and 11% of cases, respectively. In comparison, of the 17 high grade small intestinal lymphomas, 14 (82%) were associated with mucosal-invasive bacteria, serosal colonisation was evident in 10 (57%), and intravascular bacteria were observed in 5 (29%) [56]. In contrast, bacteria were not detected in any of the cases of LGAL [56]. However, it remains unclear whether invasive bacteria might trigger the development of AL or are instead opportunistically colonising the damaged mucosa after AL has developed [56]. Further prospective studies are required to establish whether lymphomatous mucosa could be permissive for bacterial colonisation in this context.

#### Chronic inflammation

It is suggested that chronic inflammation can increase the risk of developing AL, not least given that concurrent inflammatory bowel disease (IBD) has been described in up to 60% of AL cases [57–61], with many hypothesising that IBD may precede or promote digestive neoplasia [20, 38, 59, 62–66]. It has also been suggested that certain forms of human coeliac disease might actually represent low-grade intraepithelial T-cell malignant lymphoma rather than being a reactive T-cell proliferation [67, 68], and this has also been suggested for feline lymphoplasmacytic enteritis [20].

However, to date, there is no published veterinary evidence demonstrating the development of AL subsequent to chronic intestinal inflammation. In one recent study, MDR1 gene expression (MDR1, which encodes an efflux pump membrane protein) and cyclo-oxygenase 2 (COX2) were assessed in cats with chronic enteropathies, and were found to be greater in cats with LGAL than in cats with IBD [63]. It is not possible to determine if increased MDR1 expression plays a role in the aetiopathogenesis of feline IBD and LGAL but these results could explain the necessity of a more aggressive therapy in the lymphoma [63]. However, human patients with IBD show reduced expression of MDR1 in the colon and overexpression of COX2 may be associated with cell proliferation and angiogenesis [69, 70].

#### Other factors

Chronic cigarette smoke exposure and geographic factors have been suggested to promote AL but reported cases were not further subdivided based upon grade [8, 11, 14, 71]. Dietary factors have also been implicated in the development of LGAL, although this is controversial, because there is no direct evidence for it [11, 29]. Further studies are required to clarify the role played by all such factors.

## Clinical presentation

### Signalment

LGAL usually affects ageing cats (median age 13 years) [8, 28, 59, 60, 72–74], and some studies have suggested that male cats are predisposed [4, 11, 20, 40, 60]. The role of breed is less clear; to date, no specific association has consistently been found between breed and LGAL in cats, although domestic shorthair and Siamese are over-represented in some studies of AL, [59, 60, 73].

### Clinical signs

LGAL has a chronic progressive course over weeks or months, with clinical signs (in order of frequency) including: weight loss (80–90%), vomiting (70–80%), anorexia (60–70%), diarrhoea (50–65%), and to a lesser extent, icterus, splenomegaly, polydipsia/polyuria and lethargy [2, 3, 7, 8, 14, 38, 59, 61, 62, 72, 75–78]. These signs are not pathognomonic and overlap with other alimentary and indeed non-alimentary diseases. Therefore, differential diagnosis requires excluding metabolic diseases, endocrinopathies, inflammatory diseases such as feline triaditis, infectious processes, and other neoplastic diseases. Given the fact that LGAL mainly affects older cats, potential comorbidities may complicate the clinical features. However, the major diagnostic challenge faced by clinicians is distinguishing LGAL from IBD (see Table 4) and other forms of AL. It is suggested that abdominal palpation can help because, in contrast to LGAL, aggressive forms of AL are more commonly associated with abdominal mass lesions, lymphadenomegaly, peritonitis or ascites [1, 3, 7, 77]. Nevertheless, the findings of abdominal palpation do not reliably distinguish IBD and AL [2, 8, 17]. When LGAL is involved, thickened bowel loops may be identified, although this can also be a non-specific finding [8, 15]. Finally, it should be noted that abdominal palpation may also be normal in cats with LGAL [79].

### Gastrointestinal location

In LGAL, lesions are diffuse rather than localised, and can be focal or multiple, affecting any component of the gastrointestinal tract including the stomach, small intestine or large intestine as well as the mesenteric lymph nodes, liver or spleen [2, 11, 14, 38, 59, 73]. Although all sections of the gastrointestinal tract can be affected by LGAL, the most common sites include the jejunum and the ileum, followed by the duodenum [6, 20, 59]. Gastric involvement is uncommon for this type of AL (see Table 1) [1, 6, 15, 20, 59, 74, 80, 81].

**Table 1:** Segments of the gastrointestinal tract affected by low-grade alimentary lymphoma (LGAL)

Segments of the small intestine	Prevalence	Study
Duodenum	83% (10/12)	Lingard et al. [59]
Jejunum	100% (15/15)	Lingard et al. [59]
	86% (43/50)	Fondacaro et al. [20]
Ileum and ileocaecocolic junction	93% (13/14)	Lingard et al. [59]

## Diagnosis

As mentioned above, the clinical signs of LGAL are not pathognomonic making diagnosis challenging. During diagnostic investigations, metabolic diseases, endocrinopathies, infectious diseases, chronic cholangitis, pancreatitis, and exocrine pancreatic insufficiency should first be ruled out using clinicopathological testing and diagnostic imaging [79, 82]. Thereafter, LGAL should be differentiated from other gastrointestinal tract diseases such as food-responsive enteropathy (FRE) and IBD. Whilst dietary trials can usually eliminate FRE as a possible cause, distinguishing LGAL from IBD (such as lymphoplasmacytic enteritis [LPE] or eosinophilic enteritis [EE]) is more challenging because clinical signs, results of clinical pathology, diagnostic imaging findings, and even histological features can overlap [8, 11, 15, 60, 83–85]. Ultimately, more advanced diagnostic techniques such as immunohistochemistry and PCR for Antigen Receptor Rearrangement (PARR) are required to confirm the diagnosis.

### Paraclinical data

Extensive tests are often required to distinguish LGAL from other diseases, and a typical investigation would involve haematology, serum biochemistry, urine and faecal analyses, total thyroxine concentration, diagnostic imaging and intestinal biopsies. Cats should also be tested for FeLV and FIV, given the previously-reported associations with AL [75], and serum folate and cobalamin concentrations should be measured to determine the presence of malabsorption.

### Biomarkers

Biomarkers that have been used in cases of AL are summarised in Table 2. To the authors’ knowledge, no study has yet demonstrated the ability of paraclinical data to differentiate LGAL from IBD. However, failure to recognise and correct hypocobalaminaemia is known to delay clinical recovery, even when specific therapy for AL or IBD is instituted [82, 86]. Hypoalbuminaemia is reported in 70% of cats with AL, 49% with LGAL, and 77% with IBD respectively [20, 87]. The reason why hypoalbuminaemia is less common in LGAL, compared with other types of AL, is not known but might reflect the fact that mucosal integrity is preserved until later in the disease process [2, 8, 14]. In dogs, cytokine IL6 suppresses albumin synthesis and may be released by neoplastic cells [88], and this might contribute to the progressive hypoalbuminaemia observed in AL [88, 89]. Previous studies have documented the prevalence of hypocobalaminaemia in cats with gastrointestinal disease, with approximately a third of IBD cases being affected [82, 83, 87, 90–93]. Between 50 and 80% of LGAL are associated with hypocobalaminaemia (Ref.: > 290 ng/L) [2, 8, 11, 72, 75, 83, 87, 90, 94]. Not only is hypocobalaminaemia of diagnostic and prognostic significance, it can also exacerbate signs of diarrhoea, vomiting, anorexia, and weight loss, as well as causing weakness when severe.

**Table 2:** Description and prevalence of paraclinical data abnormalities reported in alimentary lymphoma (AL)

Data	Description	Paraclinical data	AL	
			Cases	Prevalence
Albumin	Biomarker of gastrointestinal protein loss [87]	Decreased [87]	LGAL	49% (21/43) [20]
Cobalamin (Vitamin B12)	Biomarker of absorption [8, 72, 83, 92, 170]	Decreased [87]	LGAL	50–80% [2, 8, 11, 72, 75, 83, 87, 90, 94]
			AL	35,3% (6/17) whose 12 LGAL [77]
Folate (Vitamin B9)	Biomarker of absorption and dysbiosis [8, 72, 83, 86, 92, 170]	Increased or decreased [82, 87]	LGAL	37% (10/27) > 21.6 ng/ml [72]
			AL	31% (4/13) < 9.7 ng/ml [83, 87]
Lactate dehydrogenase (LDH)	Biomarker of cellular necrosis [171]	Increased [172]	LGAL	47% (9/19) [172]
Fecal a1 proteinase inhibitor concentration	Biomarker of gastrointestinal protein loss [87]	Increased [87]	AL	100% (8/8) [87]
Total serum protein	Biomarker of gastrointestinal protein loss [87]	Decreased [87]	AL	100% (7/7) [87]

### Ultrasonography

**Fig. 1:**
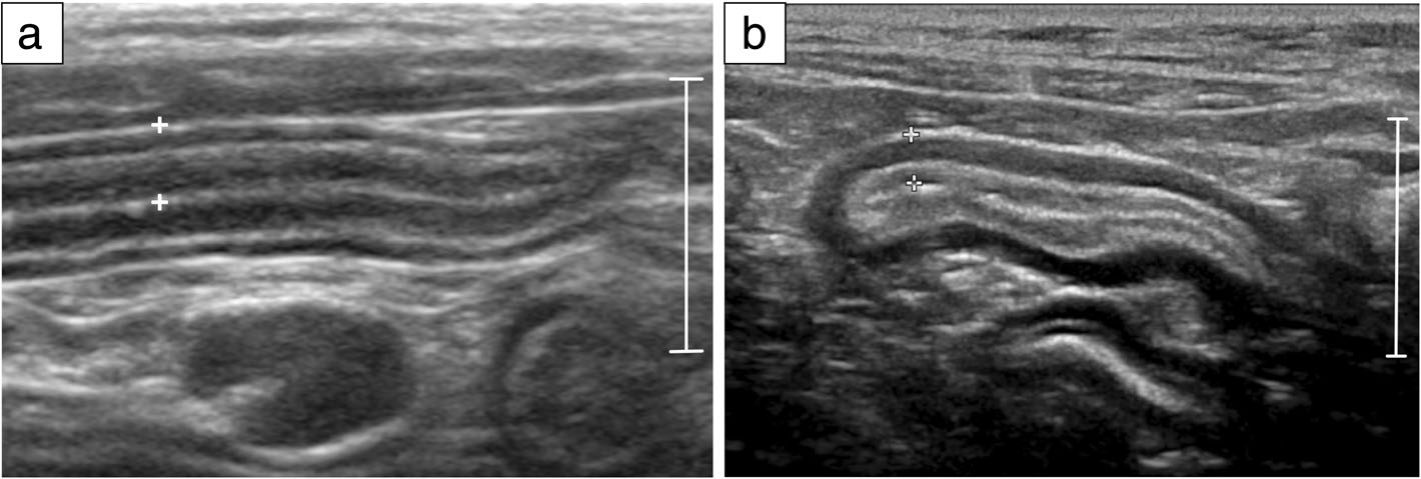
Ultrasonographic appearance of normal intestine (**a**) and low-grade alimentary lymphoma (LGAL) (**b**), longitudinal section. Note the marked thickening of the muscularis propria in the patient with LGAL (**b**) compared to a cat with a normal jejunal layering (**a**). The full thickness of both loops (between calipers) is within normal limits: normal jejunum 2.7 mm and LGAL jejunum 2.5 mm. Scale: 10 mm

**Fig. 2:**
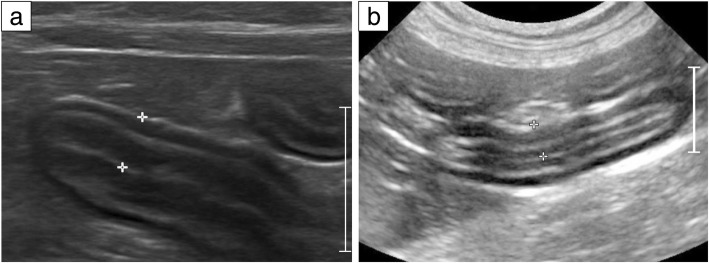
Ultrasonographic images depicting a diffuse intestinal wall thickening in low-grade alimentary lymphoma (LGAL) (**a**) and inflammatory bowel disease (IBD) (**b**). **a** Cat with advanced LGAL (duodenum): a moderate thickening of the muscularis propria is observed. Full-thickness (between calipers): 3.7 mm. **b** Cat with IBD, eosinophilic enteritis (small intestine): note the similar ultrasonographic appearance of the intestine compared to LGAL aspect. Full thickness (between calipers): 3.9 mm. Scale: 10 mm

Abdominal ultrasonography allows reliable detection of focal or diffuse thickening as well as loss of the normal layered appearance of the gastrointestinal wall (Fig. 1) [15, 95]. In addition, this imaging modality may reveal other LGAL-associated lesions, such as mesenteric lymphadenomegaly, hepatomegaly and splenomegaly [2]. The most common ultrasonographic finding is a diffuse thickening of muscular layer in the small bowel, and this characteristic is observed in 50–80% of cases [15, 59, 77, 95–97]. Unfortunately, given that this is also commonly observed in feline patients with IBD [98], ultrasonographic distinction between LGAL and IBD is not possible (Fig. 2 and Table 3). For example, in one study involving 22 cats with FRE, 17 with IBD and 17 with AL, there were no significant differences in ultrasonographic findings between the three groups [82]. Moreover, ultrasonography is less sensitive for the detection of small lesions of the gastrointestinal tract than is endoscopy [2]. Therefore, LGAL can never be excluded on the basis of a normal ultrasonographic examination and histological analysis is always required to make a definitive diagnosis [2, 95].

**Table 3:** Comparison of ultrasonographic features observed in low-grade alimentary lymphoma (LGAL) and inflammatory bowel disease (IBD)

Ultrasonographic parameter	LGAL	IBD
Gastrointestinal wall thickness	Muscularis propria frequently thickened [8, 15, 59, 82, 95, 101]	Muscularis propria frequently thickened in eosinophilic enteritis (EE), possibly increased in lymphoplasmacytic enteritis (LPE) [15, 78, 82, 84, 95, 101]
Size of mesenteric lymph nodes	Lymphadenomegaly (> 5 mm) frequent but not systematic [8, 82, 95, 101, 102]	Lymphadenomegaly frequent but not systematic [78, 82, 95]
Gastrointestinal intramural masses	Rare but possible [75]	Rare but possible [75]
Stratification and architecture	Normal [8, 59] to modified [61, 82, 101]	Normal to modified [82, 95]
Motility	Normal to reduced [95]	Normal to reduced [95]
Intussusception	Rare but possible [2, 75]	Very rare but possible [2, 75]
Liver appearance	Hypo- or hyperechogenicity possible, lobular pattern if liver involved [2]	Non specific [2]
Pancreas	NA	Changes suggestive of pancreatitis (pancreatic hypoechogenicity, peripancreatic hyperechoic fat) [78, 173]

*NA* not available

Thickening of the gastrointestinal wall is the most common lesion observed in AL (Fig. 2) [2]. However, although reference measurements for wall thickness have been reported in cats, clear diagnostic cut-points for either LGAL or IBD have not been reported [95, 99]. Potentially of more utility, is the identification of a thickened muscularis propria, which might be twice as thick as that seen in healthy cats [15]. Typically, the ratio of muscularis width to submucosal width in calculated, with measurements ≥0.5 being considered abnormal [15, 95]. This feature is reported to be common in feline LGAL [15, 82, 95], although it is not pathognomic because it is also reported in IBD [15, 82], particularly in eosinophilic enteritis (EE) [100]. That said, older cats with ultrasonographic evidence of muscularis propria thickening are more likely to have AL versus IBD [78, 95].

Enlarged mesenteric lymph nodes are another common ultrasonographic finding [8, 82, 95, 101, 102]. In one retrospective study, mesenteric lymphadenomegaly was reported in 6 out of 10 AL cases, and 2 out of 12 IBD cases [77]. Mesenteric lymph node thickness can vary, with the average being 4 to 6 mm for jejunal lymph nodes and 5 mm for ileo-caecal lymph nodes [15, 99]. In AL, changes in lymph node size can be highly variable, ranging from minimal to markedly enlarged, although, in recent studies, moderate enlargement is most likely [2, 59, 77, 95, 96]. Mesenteric lymph node enlargement was reported in 17 LGAL cases in one study, with a mean diameter of 15.9 mm (range 6.5–30 mm) [59]. In a more recent study, colic lymph nodes in LGAL cats were reported to be 1.6-fold larger in size than those from healthy cats whereas jejunal lymph node hypertrophy was not systematically associated with LGAL [15].

### Endoscopy and coeliotomy

Gastrointestinal endoscopy is an important investigative tool in feline gastroenterology providing the ability to access the stomach, duodenum, distal ileum and colon, as well as allowing direct visualisation of the mucosa [75, 103]. Experienced endoscopists can often reach the proximal jejunum [103, 104]. It is a minimally invasive and rapid technique allowing macroscopic lesions to be described and multiple intestinal biopsy samples to be collected, albeit exclusively from mucosa or submucosa [15, 20, 72, 105]. Mucosal lesions that cannot be characterised by ultrasonographic examination may be identified by endoscopy [75, 106, 107]. Macroscopic differentiation between inflammatory lesions (congestion, oedema, mucosal fibrosis) and LGAL lesions is not possible by endoscopic examination [20, 107]. In addition, the jejunum and the proximal part of the ileum, which are the most frequent locations of LGAL, are often not accessible by endoscopy [105]. Evans et al. suggested exploratory cœliotomy may be preferable to endoscopy [77]. However, in their study involving 10 AL and 12 IBD, duodenal assessment was limited in half the cats and biopsies were performed blindly in 8 of the cats which might have contributed to the poor sensitivity of endoscopic biopsies. By obtaining endoscopic specimens of the jejunum and ileum, laparoscopy may improve the diagnostic sensitivity and may therefore be a minimally invasive alternative to exploratory cœliotomy [77].

Although exploratory cœliotomy is more invasive than endoscopy, all segments can be inspected, full-thickness biopsies (mucosa, submucosa, muscularis and serosa) can be taken from various sites (stomach, duodenum, jejunum and ileum) [3, 4, 11, 15, 59, 75], and biopsies of other organs (e.g. peripheral lymph nodes, liver, and pancreas) if indicated [99]. The technique is generally safe with post-operative complications being uncommon [108]. Indeed, there was no clinical evidence of postoperative leakage from the biopsy sites and a low incidence of post-operative complications in published reports involving 70 AL cases where full-thickness gastrointestinal biopsies were taken [75, 76]. Unfortunately, regardless of method used for biopsy sampling, diagnosis remains challenging because of the frequent lack of gross changes in cases of LGAL. Multiple systematic biopsies of all the digestive areas are therefore recommended [14, 61, 79, 107].

### Cytology and histology

Both cytological and histological assessment can be used to aid in the diagnosis of AL in cats. Whilst cytology samples can be easier to collect, results may be unreliable, particularly for LGAL, where the rate of false-negatives for mesenteric lymph nodes can reach 50% [59, 60, 75, 107, 109–111]. Few intra-abdominal lymph node cytology results are positive for LGAL, possibly because the neoplastic lymphocyte population mainly comprises small lymphocytes, which would not be considered to be unusual in a lymph node [60]. Conversely, aspiration cytology of focal intestinal wall masses or enlarged mesenteric lymph nodes are considered adequate to diagnose HGAL or LGLL [82, 107].

**Fig. 3:**
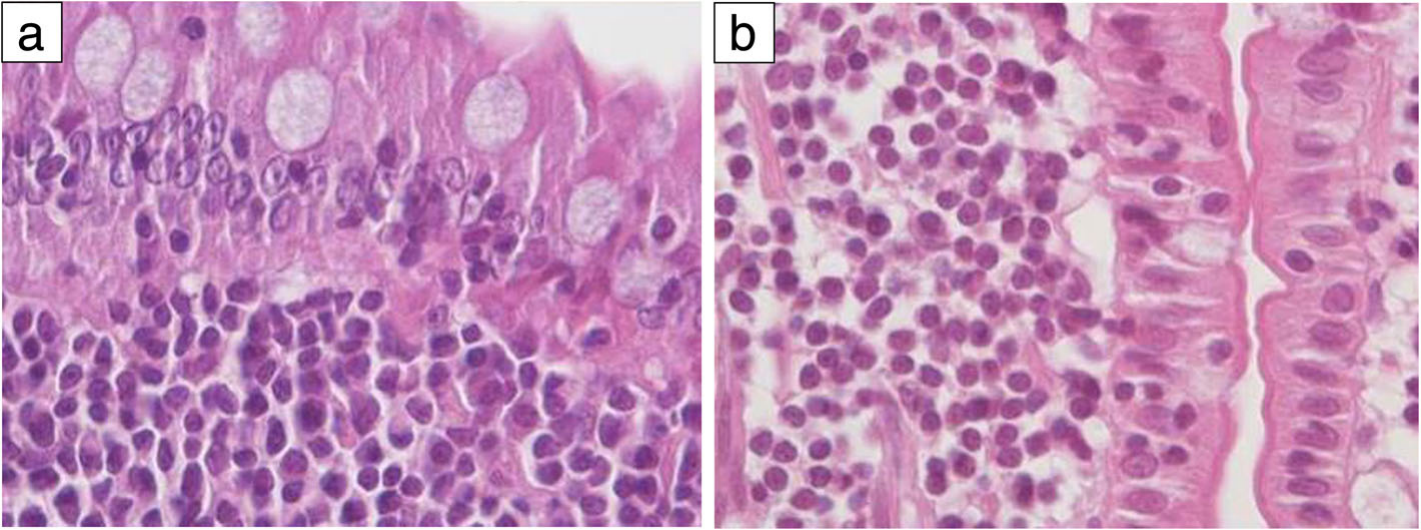
Histological features of low-grade alimentary lymphoma (LGAL) and plasmacytic enteritis (haematoxylin-eosin-staining, 400X). **a** LGAL: monomorphic dense infiltrate of small lymphocytic cells with discrete nuclear atypia; some plasma cells are present. **b** Plasmacytic enteritis: less compact infiltrate of small lymphocytes with dense nucleus; more plasma cells are present

Histological examination of the small intestine is important in establishing a diagnosis of AL [1, 28, 97, 101, 109, 112], and it is advisable to define clinical features according to the guidelines of the World Small Animal Veterinary Association Gastrointestinal Standardization Group [104, 113]. LGAL lesions are characterised by an infiltrate of neoplastic lymphocytes of small-to-intermediate size and involving the epithelium and lamina propria of the villus (Fig. 3); infiltration of the submucosa and muscularis is also frequent [6, 58, 60, 75]. Since the MALT includes the largest population of lymphoid and accessory immune cells in the gastrointestinal tract, it is the primary site of neoplastic proliferation [6, 114]. Differentiation between inflammatory and neoplastic infiltrates is often challenging because the histological features of LGAL and IBD (especially lymphoplasmacytic enteritis) can be similar (Fig. 3) [58, 82]. However, amongst the criteria to distinguish LGAL from IBD, epitheliotropism, defined as the characteristic homing of neoplastic T-cells to the mucosal epithelium [60], is arguably most important, and in particular the presence of intraepithelial nests and plaques [6, 19, 60, 61, 79]. An intraepithelial nest is defined as 5 or more small T-lymphocytes clustered within the villous epithelium, and an intraepithelial plaque as 5 or more adjacent epithelial cells obscured by infiltrates of small T-lymphocytes [61, 85]. Mild cases may be limited to purely intra-mucosal infiltration of small T-cells into the epithelium layer [60]. Two studies found that the majority of the intraepithelial lymphocytes in healthy and specific pathogen-free cats were CD3^+^ [114, 115]. Whilst epitheliotropism is suggestive of LGAL rather than IBD, its absence cannot be used to exclude the condition because it is not observed in all digestive T-cell lymphomas [61].

Therefore, intraepithelial lymphocytes are an important cell population to study during histological assessment of feline gastrointestinal biopsies, and are usually of T-cell phenotype [6, 60]. In healthy cats, there are many more intraepithelial lymphocytes in the villous epithelium than in the crypt epithelium in all regions of the small intestine, and this is also for cats with IBD, AL, and epitheliotropic intestinal malignant lymphoma (EIL) [60, 115]. However, there are usually markedly more intraepithelial lymphocytes in cats with EIL than either healthy cats or cats with IBD [60]. Further, the common occurrence of epitheliotropism in small intestinal T-cell lymphoma has been described [6, 57, 60], with one study demonstrating that 62% of mucosal T-cell lymphoma of small-cell type had characteristics of EIL. Epitheliotropism is also an important feature of HGAL, occurring in 58% of transmural T-cell lymphoma and in some T-cell LGLL of the small intestine [6, 114, 116].

Inter-observer variability can occur and histology must be correlated with clinical status and outcome [117, 118]. Moreover, a discrepancy between the perceived lower number of intraepithelial lymphocytes based on HE stains and the greater number of intraepithelial lymphocytes based on CD3 stain has been noted, possibly because of the difficulty in differentiating them from enterocytes in HE-stained sections [60, 115]. In addition to the described changes in lymphocyte populations, concurrent histological abnormalities in neoplastic regions include lymphoid microabscesses in the mucosal epithelium, villous blunting and fusion, increased plasma cell infiltrate within the lamina propria and eosinophil infiltrate into the lamina propria [60].

### Immunohistochemistry

Immunohistochemistry has become an increasingly important molecular technique to improve the diagnosis of AL, especially to aid in the differentiation of LGAL and IBD [10, 15, 62, 77, 103, 106, 119]. Since it can often be performed on formalin-fixed and paraffin-embedded (FFPE) biopsies, it must be readily performed as a complementary technique to standard histological examination. With IHC, specific monoclonal antibodies are applied that recognise antigenic determinants (epitopes), thereby enabling microscopic detection of differentiation and proliferation biomarkers [10, 62, 111]. The most commonly-used antibodies include anti-CD3 to detect T-lymphocytes [6, 10, 39, 60–62, 79, 120], coupled with anti-CD79a, anti-CD20 and anti-BLA36 to detect B-lymphocytes [39, 60–62, 72, 103], MAC387 highlights macrophages [10, 62], and anti-CD57 is used to detect natural killer (NK) cells [6]. Moreover, the percentage of proliferating cells can also be determined immunohistochemically by detecting Ki67 expression [10]. Ki67 is expressed in the nucleus during all phases of the cell division cycle, with its concentration increasing during the cell cycle, becoming maximal expression in M-phase and then totally disappearing after mitosis [10, 121, 122]. However, whilst useful, the authors are not aware of any threshold value that reliably discriminates either between the different feline AL subtypes, or IBD [10]. Other specific feline antibodies (anti-CD4, anti-CD8α, anti-CD8β) can be used to detect additional epitopes and define the immunophenotype of lymphomas, but their routine clinical application is limited by the fact that formalin-fixed samples cannot be used as frozen sections are required [10].

**Fig. 4:**
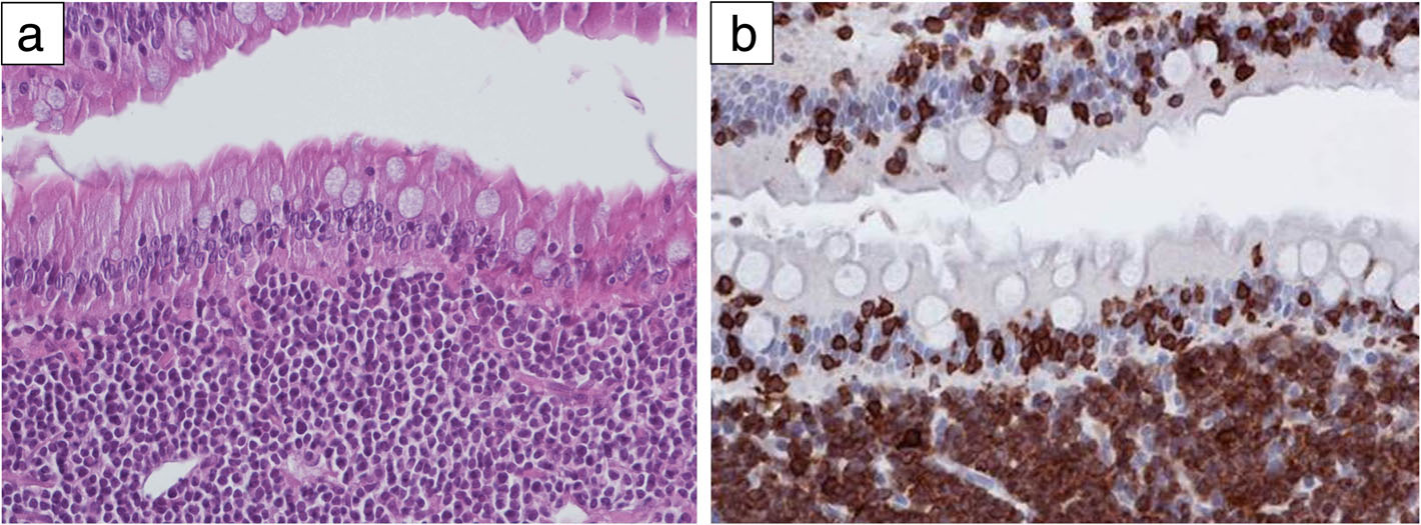
Histological (**a**) (haematoxylin-eosin-staining, 200X) and immunohistochemical (**b**) (anti-CD3, immunoperoxydase, 200X) features of low-grade alimentary lymphoma (LGAL) showing dense infiltration of the lamina propria composed of a mixture of small CD3^+^ T lymphocytes and plasma cells, with epitheliotropism (small intestine biopsies)

LGAL is of T-cell phenotype in 90% of the cases, staining positively with anti-CD3 antibody (Fig. 4), with a B-cell phenotype observed in the remaining 10% of cases [6, 8, 10, 11, 58, 63, 75, 97]. However, there is increasing evidence that some human and canine T-cell lymphomas co-express the B-cell marker CD20 [123–125]. In a three-case series reported by Nolan and Kiupel (2018), the majority of neoplastic CD3^+^ lymphocytes expressed CD20 but not Pax5. In all cases, PARR testing demonstrated clonal rearrangement of the T-cell receptor gamma gene, suggesting a diagnosis of CD3^+^, CD20^+^ enteropathy-associated T-cell lymphoma, large cell type [80]. This explains why the PARR is most suited to detecting clonally expanded lymphocyte populations, rather than determining phenotype [85].

Moreover, human peripheral T-cell lymphoma may exhibit aberrant expression of CD20 [123], and a subset of T-lymphocytes with low-expression of CD20 (known as CD20^dim^ T-lymphocytes) has been detected in normal peripheral human blood [126]. It is not currently known whether CD20 expression in canine lymphoma reflects aberrant expression or whether the neoplastic lymphocytes originated from a subpopulation of normal CD20^dim^ T-cells. Finally, in a recent study involving 40 T-cell AL, 5 B-cell AL and 8 IBD, T-cell lymphomas in particular were characterised by a high percentage of Bcl-2 (B-cell lymphoma gene 2) labelled cells, whilst B-cell lymphomas varied more widely in the number of positively-labelled cells [19]. Further studies are required to clarify the exact phenotypes of LGAL, although this has been hampered by the limited number of antibodies available for IHC studies.

### Clonality testing

In conjunction with other diagnostic techniques, clonality analyses have been increasingly used in veterinary pathology to differentiate reactive and neoplastic lymphoid proliferations, for example differentiating IBD from LGAL [6, 112, 127]. This diagnostic tool was first reported for feline intestinal T-cell lymphoma by Moore et al. in 2005 [57], with the most commonly reported approach being PARR. This technique is based on PCR targeting of the CDR3 region of T-cell receptor gamma (TCRγ) for T-cells and immunoglobulin heavy chain genes (IGH) for B-cells [57, 58, 85, 95, 111, 127–129], and can detect clonally expanded lymphocyte populations. However, PARR cannot determine lymphocyte phenotype and, therefore, it complements rather than replaces the use of immunohistochemistry. This is because neoplastic lymphocytes may have clonal rearrangement of either or both the T- or B-cell antigen receptor genes, regardless of phenotype [57, 61, 85, 130]. For example, cases of mucosal T-cell lymphoma of small cell type may show clonal rearrangement of the IGH gene. This is termed *cross-lineage rearrangement* and can be an effect of malignant transformation but causes remain to be elucidated [61, 85, 131, 132]. *Cross-lineage rearrangement* has previously been reported in both human and canine T-cell malignancies [133, 134].

More than 90% of LGAL exhibit a clonal or oligoclonal TCRγ gene rearrangement, whereas IBD usually displays a polyclonal pattern [6, 57]. Moreover, the diagnostic sensitivity and specificity of PCR-based clonality assays for routine diagnosis of feline TCRγ gene rearrangement are currently close to 90% [6, 57, 128, 135]. As a result, Sabattini et al. recommended the assessment of clonality in duodenal endoscopic biopsies to increase the detection sensitivity to distinguish between LGAL and IBD [112]. Taken together, these data suggest that clonality testing should be integrated in the routine diagnosis work-up of LGAL. Initially developed on frozen samples, molecular clonality testing can now be performed on formalin-fixed and paraffin-embedded (FFPE)-derived samples [136], making this assay more easily accessible. The average sensitivity for diagnosis of feline AL was highest (0.83), intermediate (0.78), and lowest (0.72) for using histology and IHC and clonality, histology and IHC, and histology alone, respectively [61, 107].

Even where there is agreement between histological findings and clonality analysis, clinical, morphological, and immunophenotypic data should ideally be integrated with clonality analysis in order to reduce the chances of a misdiagnosis [6, 19, 61, 82, 127]. Cross-lineage rearrangement of the IGH gene was detected in at least 9% of cases diagnosed as T-cell AL [85, 129]. Moreover, a polyclonal rearrangement of the TCRγ gene can also be observed. Reasons for this pattern include the presence of inflammatory T-cell infiltrates within the evaluated tissues which then mask the clonal T-cell population, or the exclusive amplification of the TCRγ gene of resident or inflammatory T-cell populations of the intestine [57, 85]. Conversely, detection of a clonal lymphocyte population is not always indicative of neoplasia, since this can sometimes be seen as a response to pathogens and concurrent malignancies [61, 85]. In the study of Kiupel et al., involving 47 AL and 16 IBD, one case had clonal population of B-cells and a final diagnosis of IBD was made, when histomorphology and immunophenotyping results were taken into consideration. Amongst ten cats with an oligoclonal population of T-cells, one case was diagnosed as IBD [61]. Moreover, high percentage of clonality has been associated with marked inflammatory process in humans [137].

That said, whilst clonality testing can provide additional information on a particular case, the use in diagnosis has not yet been critically reviewed and properly validated. Therefore, for now, clinicians should be cautious about reclassifying cats previously diagnosed with IBD to lymphoma on the basis of PARR, and a combination of conventional histopathology and immunohistochemistry (IHC) remains the current gold-standard test.

### Staging

To our knowledge, the staging criteria classically defined for other types of lymphoma cannot be reliably used to classify LGAL [3, 5, 20, 38].

## Distinguishing low-grade alimentary lymphoma from inflammatory bowel disease

As discussed above, a major challenge of the clinician is to distinguish LGAL from IBD given that their clinical presentations overlap. Table 4 provides a summary of the two conditions comparing aetiology, signalment, clinical signs, and diagnosis.

**Table 4:** Comparison of aetiology, epidemiology, and clinical features in cats with low-grade alimentary lymphoma (LGAL) and inflammatory bowel diseases (IBD)

	LGAL	IBD
Aetiology	Currently unknown	Currently unknown, multifactorial disease, though many factors implicated including genetic factors and enteric bacteria or protozoa [3, 11, 52, 63]
Age	Mainly older cats [4]	Any age [63, 78, 79, 82]
Breed	No breed predisposition [59]	Domestic shorthair and longhair, Persian, Siamese predisposed [63, 78, 79, 87]
Gastrointestinal locations	Any but jejunum and ileum most common (90%) [58, 59, 119]	Any but duodenum and ileum most common (70–90%) [58, 59, 119]
Clinical signs	Weight loss, vomiting, anorexia, diarrhea, lethargy [8, 63, 79]	Weight loss, vomiting, anorexia, diarrhea, lethargy [8, 63, 79]
Biomarkers		
Albumin	Decreased (49%)	Decreased (77%) [87]
Total proteins	NA	Increased (18%) [78]
Cobalamin	Decreased (50–80%) [2, 8, 11, 75, 82, 83, 87, 90, 94]	Decreased (18–47%) [78, 82, 87]
Folate	Increased (37%) [72]	Increased (22%) [87]
LDH	Increased (47%) [172]	Increased (26%) [172]
ALP and ALT	NA	Increased (23%) [78]
fPLI	NA	Increased (18%) [78]
Phosphate	NA	Decreased (47%) [78]
Ultrasonography	Muscularis propria frequently thickened [8, 15, 59, 95, 101]; mesenteric (i.e. jejunal) lymphadenomegaly frequent [95]; gastrointestinal intramural masses rare [75]; stratification, architecture and motility normal to modified [95]	Muscularis propria frequently thickened in eosinophilic enteritis (EE), and occasionally in lymphoplasmacytic enteritis (LPE) [15, 78, 84, 95, 101]; mesenteric lymphadenomegaly frequent [78, 95]; gastrointestinal intramural masses rare [75]; stratification architecture, and motility normal to modified [95]
Histological features and immunohistochemistry	Diffuse infiltration by monomorphic neoplastic T-cells [6, 8]	Polymorphic inflammatory infiltrate of lymphocytes, plasma cells (LPE), neutrophils, eosinophils (EE), and macrophages [75, 84, 174]
Clonality test	Clonal population of lymphocytes [10, 15, 62, 95]	Polyclonal population of lymphocytes [10, 15, 95, 174]

*NA* not available

## Treatment

### Chemotherapy

Systemic chemotherapy is generally considered to be the most effective treatment for AL [2, 39], although protocols have not been frequently described [3]. Compared with other forms of lymphoma, less intensive chemotherapy protocols have been suggested [20], with the most common protocols involving oral administration of both glucocorticoids (prednisolone or dexamethasone) and chlorambucil (Table 5) [20, 59, 72, 97]. The optimal duration of chemotherapy has not been determined and, because these drugs are well tolerated, treatment is rarely discontinued. In one study involving 56 cases of feline small cell lymphomas (37 AL), all cats were treated with glucocorticoid and chlorambucil with discontinuation of treatment recommended at one year if complete clinical response was documented. Subsequent reintroduction as rescue chemotherapy appears to be just as effective as continued administration in cats and median overall survival times for cats with AL was 1148 days [96].

**Table 5:** Description of chemotherapy protocols based on Chlorambucil (PO) and Prednisolone (PO) administration in low-grade alimentary lymphoma (LGAL) [20, 59, 72, 97]

Study	Stein et al. [97]	Lingard et al. [59]	Kiselow et al. [72]	Fondacaro et al. [20]
Number of cases	28	12	41	29
Prednisolone	1–2 mg/kg PO q24h^b^	3 mg/kg PO q24h, tapering to 1–2 mg/kg once in remission	5 mg/cat PO q12-24 h	10 mg/cat PO/cat/day
Chlorambucil	20 mg/m^2^ PO q2wk^c^	15 mg/m^2^ PO q24h for 4d q3wk	2 mg/cat PO q48h	15 mg/m^2^ PO q24h for 4d q3wk^d^
Number responding	27 (96%)	NA	37 (95%)	NA
Complete remission rate	NA	NA	22 (56%)	20 (69%)
Median remission time^a^	786 days	505 days	897 days	615 days
Median survival time	NA	513 days	704 days	510 days

*NA* not available

^a^Remission time in days for cats displaying a complete response; ^b^Two cats received dexamethasone, initially at immunosuppressive dosages and then at dosages that were gradually tapered over the course of 3 weeks; ^c^Because of client preference, two cats were switched to 20 mg/m^2^ chlorambucil orally q3wk; ^d^Twelve of the 20 cats that achieved CR received cyclophosphamide 225 mg/m^2^ PO q3wk, once they were out of remission

### Rescue protocol

Other chemotherapy protocols can be used if relapse is experienced, with examples including: cyclophosphamide and prednisolone; cyclophosphamide, vincristine and prednisolone (COP protocol) +/− L-asparaginase and doxorubicin (VELCAP-C); lomustine PO with or without corticosteroid [2, 4, 7, 39, 82]. In the study of Stein et al., involving 28 cats diagnosed with LGAL and treated with a combination of chlorambucil and glucocorticoids, seven of the nine cats with relapsed disease were treated with a rescue protocol of cyclophosphamide (PO 200–250 mg/m^2^ given on days 1 and 3 q2wk) and prednisolone (5 mg q48h). The response rate was 100% based on resolution of clinical signs and normal abdominal palpation. Three cats died of unrelated diseases, three cats were lost to follow-up, and the final cat relapsed 241 days after starting the rescue protocol [97]. However, to the authors’ knowledge, there are no studies comparing the outcome of cats with LGAL treated by other recue protocols, mainly because studies include all subtypes of AL. Interestingly, one cat in the study of Lingard et al. did not respond to a multi-agent protocol, but entered long-term remission with oral administration of prednisolone and chlorambucil, suggesting that LGAL may be sensitive to alkylating agents such as Chlorambucil [59].

### Surgical resection

Given that LGAL lesions are usually diffuse, surgical resection is rarely indicated as a means of managing the disease. In rare cases where an obstructive mass lesion is present, partial resection could be considered as part of the treatment [107, 138]. The main disadvantage of surgical resection is the risk of post-operative complications [76, 139], which can include dehiscence, typically occurring between 2 and 5 days after surgery [75]. Nevertheless, in one study involving 20 cases of intermediate−/high-grade AL where surgical resection of disease was performed prior to CHOP-based chemotherapy, no post-operative surgical complication occurred [138]. Cats with AL do not appear to be at high risk of post-operative complications after full-thickness gastrointestinal surgery [138, 139].

### Adjuvant therapy

In addition to chemotherapy, a highly digestible diet is typically recommended, with appetite stimulants (most commonly mirtazapine 3.75 mg/cat PO, every 3 days) used in cats with partial or complete anorexia [75, 107]. A recent paper described the use of growth hormone secretagogues, such as capromorelin, for appetite stimulation in cats [140]. Capromorelin was already shown to increase food intake or weight gain in dogs and humans [141, 142]. Treatment with capromorelin at 6 mg/kg once daily for 91 days in 8 healthy laboratory cats resulted in increased body weight and a greater mean food consumption compared to the 4 placebo-treated cats. The optimal clinical dose of capromorelin in cats has yet to be confirmed, although no serious adverse events were observed in the recent study [140].

Cats with concurrent hypocobalaminaemia are usually treated with supplemental cobalamin (e.g. 250 μg/cat SC once weekly for at least 6 weeks), although there is no clear consensus on doses and duration [72, 82, 86, 87]. The clinical benefits of prebiotics, probiotics and gluten-free diet as adjuvant therapies in LGAL have not yet been proved and need to be investigated in randomised studies [107].

## Prognosis

In contrast to HGAL and LGLL, prognosis for LGAL is good with a high remission rate when the treatment is carried out over several months or years [13, 14, 59, 75, 82]. Most causes of death include relapse, comorbidities, or euthanasia in accordance with the owner’s request [20, 59, 107]. Initial response to chemotherapy seems to be the most significant prognostic indicator [5, 20, 40, 143–145], with the presence of lethargy, vomiting and anorexia at initial diagnosis also considered to be negative prognosis factors [59]. However, no association has been observed between the response to LGAL treatment and a variety of other factors including: age, weight, sex, type and duration of clinical signs, presence of extra intestinal lesions, and decreased concentrations of folate, cobalamin and plasma total protein [38, 72, 75, 120]. It remains unclear as to whether, in the long-term, LGAL can progress to more aggressive forms of AL.

## Discussion

### A model for human indolent T-cell lymphoproliferative disorders of the gastrointestinal tract?

Within the “One-Health” concept, the domestic cat is considered to be a good model for comparative biomedical research. Indeed, naturally-occurring feline cancers offer opportunities for comparative and translational advances that could be of mutual benefit for both human and veterinary oncology [146–148]. Therefore, as well as improving diagnostic tools for feline LGAL, identifying aetiological factors in cats may ultimately be beneficial to human patients with indolent digestive lymphoma.

#### Human indolent T-cell lymphoproliferative diseases of the gastrointestinal tract

Human primary gastrointestinal lymphomas are very rare, accounting for less than 5% of all non-Hodgkin lymphomas (NHL) yet representing the largest group of primary extranodal NHL, with approximately 25% of cases occurring in the gastrointestinal tract [19, 149]. They are predominantly located in the stomach (50–60%), whereas intestinal lymphomas are less common and affect the small and large bowel in 20–30% and 10–20% of cases, respectively [150–152]. Amongst primary intestinal T and NK-cell lymphoma, indolent intestinal T-cell lymphomas are newly described forms of low-grade, diffusely infiltrating T-cell lymphomas.

Indolent T-cell lymphoproliferative diseases (LPD) of the gastrointestinal tract have been described as clonal T-cell proliferations, with an indolent clinical course after long-term monitoring [153]. These diseases are very rare and have only been reported as sporadic cases or in small case series [154–161]. Patients commonly present with chronic diarrhoea, weight loss, malnutrition, abdominal pain or rectal bleeding. In some cases, autoimmune diseases may develop, for example coeliac disease or autoimmune enteropathy [154–156, 162]. Macroscopic findings include villous atrophy, mucosal erythema, erosions or small ulcerations and, occasionally, small polyps without mass lesions [149]. Patients usually have multiple lesions along the gastrointestinal tract, most commonly in the small intestine and colon [149]. On histological examination, the lamina propria is densely infiltrated with a monomorphic population of small lymphoid cells. Severe intestinal villous atrophy, caused by the lamina propria infiltration, is commonly observed, but there is usually no evidence of epithelial destruction [149]. Tumour cells are CD3^+^, either CD4^+^ (more commonly) or CD8^+^, or in rare instances CD4^−^/CD8^−^, whilst those expressing CD8 have a cytotoxic profile (TIA1^+^, granzyme B^±^) [153, 154, 156, 163]. Usually, CD56, CD30, CD103 and Epstein-Barr virus are not detected, whilst the Ki67 proliferation index is low e.g. in the range of 5–10% [153, 154, 156, 163]. Tumour cells display TCRγ or β gene rearrangements and the TCRβ-chain is expressed on the surface in most cases, most notably in CD4^+^ cases [153, 154, 156, 163].

The condition is clinically indolent, and most patients are still alive with a persistent disease after several years of follow-up [153, 154, 156, 163]. Nevertheless, three cases have been reported where patients died from aggressive T-cell lymphoma several years after being diagnosed [154]. In addition, conventional chemotherapy does not lead to durable clinical and histological responses in such patients and has been associated with high toxicity [153, 154, 156, 163].

Little is known about the mechanisms involved in the pathogenesis of indolent LPD of the gastrointestinal tract. Given the coexistence of autoimmune or inflammatory diseases, it has been speculated that immune dysregulation plays a role in disease pathogenesis [153, 154, 156]. Environmental factors such as diet or infectious agents (e.g. viruses, bacteria) could also be involved in the development of this disease. Human herpes virus 6 (HHV6) was detected in the small intestinal biopsies of one patient by PCR analysis and two patients had positive serology for human T-lymphotropic virus type 1 (HTLV1) at the time of diagnosis, but with no evidence of viral integration in the intestinal biopsies [156]. In addition, no specific disease-associated chromosomal or genetic alterations have been uncovered so far. To date, the origin of indolent digestive T-cell LPDs remains unknown. In a subset of cases, there is expression of CD103, suggesting that such LPDs originate from a mucosal T-cell precursor, although further characterisation would be required [164].

#### Comparison of feline low-grade alimentary lymphoma with human digestive T-cell lymphoma

The main characteristics of each subtype of human digestive T-cell lymphoma are presented in Table 6.

**Table 6:** Pathological features of human digestive T-cell lymphoma [25, 153, 164, 165, 175, 176]

	Clinical features	Histology	Immunophenotype	Outcome
Indolent digestive T-cell lymphoproliferative disease	Diarrhoea, abdominal pain	- Crypt hyperplasia, variable degrees of villous atrophy - Non-destructive superficial infiltrate of small uniform T-cells mostly lamina propria-based - Infiltration into submucosa observed in some cases - No evident major epitheliotropism	CD3+, CD8+ or CD4+, CD2+, CD5+/−, CD7+/−, CD30-, CD56-, TCRαβ+	Indolent chronic relapsing course
Enteropathy associated T-cell lymphoma (EATL)	Overt or silent gluten-sensitive enteropathy	- Crypt hyperplasia, villous atrophy - Pleomorphic medium- to large-sized neoplastic lymphocytes with transmural infiltration - Presence of other mixed inflammatory cells such as histiocytes and eosinophils - Intraepithelial lymphocytosis present in non-tumoral mucosa and in epithelium distant from the main mass	CD3+, CD5-, CD8−/+, CD56-, CD103+, often CD30+, cytotoxic phenotype +/−, TCR αβ + (usually)	Aggressive
Monomorphic epitheliotropic T-cell lymphoma	Occurs without a history of coeliac disease	- No crypt hyperplasia, possible villous atrophy - Monomorphic infiltrate with epitheliotropism - Transmural infiltration - No associated inflammatory background	CD3+, CD5-, CD4-, CD8+, CD56+, cytotoxic phenotype, CD30-, TCR γδ + (usually)	Aggressive

**Fig. 5:**
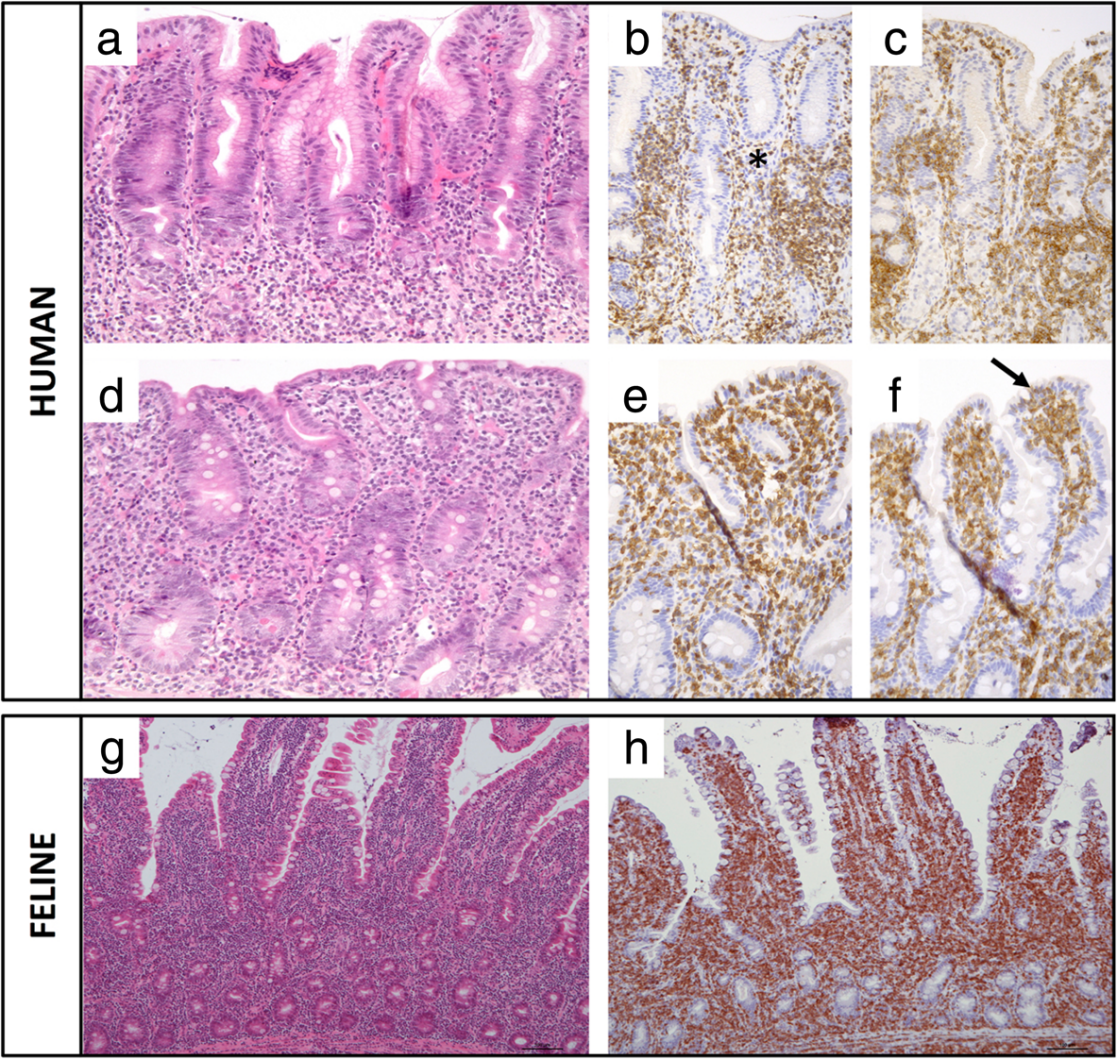
Comparison of histological and immunohistochemical features of feline low-grade alimentary lymphoma (LGAL) and human indolent digestive T-cell lymphoproliferative disease (LPD). Top Panel: Human indolent CD4^+^ T-cell lymphoproliferative disease of the gastrointestinal tract. Biopsies of the antrum (**a**, **b**, **c**) and duodenum (**d**, **e**, **f**) show important CD3^+^ (**b** and **e**) and CD4^+^ (**c** and **f**) lymphoid infiltrate into the lamina propria (asterisk), mostly composed by small lymphocytes. Epitheliotropism is mostly absent, with however focal exceptions such as small CD4+ T-cells localized here in the duodenal epithelium (arrow). Bottom Panel: Feline T-cell low-grade alimentary lymphoma. Biopsies of the jejunum show epitheliotropic lymphocytic infiltrate involving the lamina propria (**g**), exhibiting a CD3^+^ phenotype (**h**)

Although it has been suggested that the feline LGAL most closely resembles enteropathy-associated T-cell lymphoma (EATL; previously designated type I EATL) or monomorphic epitheliotropic intestinal T-cell lymphoma (MEITL; previously designated type II EATL) [6], the authors contend that it has more characteristics in common with indolent digestive T-cell LPDs in humans, according to the latest WHO classification [165]. Clinical, histological and phenotypic features of feline LGAL and human indolent digestive T-cell LPDs are compared in Table 7 and Fig. 5 [149, 153–156, 159].

**Table 7:** Comparison of feline low-grade alimentary lymphoma (LGAL) and human indolent digestive T-cell lymphoproliferative disease (LPD)

Data	Feline LGAL	Human indolent digestive T-cell LPD
Epidemiology	Frequent, increasing prevalence over the last decade	Very rare
Clinical signs	Non-specific weight loss, vomiting, anorexia, diarrhoea	Non-specific weight loss, diarrhoea, abdominal pain, digestive bleeding, malnutrition
Gastrointestinal localisation	Multiple lesions affecting all the gastrointestinal tract; small intestine as main involvement	Multiple lesions affecting all the gastrointestinal tract; small intestine as main involvement
Histology	Monomorphic population of small- to intermediate-sized T-lymphocytes; infiltration of neoplastic T-cells in villi and lamina propria; moderate villous atrophy; crypt hyperplasia	Monomorphic population of small- to intermediate-sized T-lymphocytes Infiltration of neoplastic T-cells in villi and lamina propria; villous atrophy (often severe); crypt hyperplasia; erythema of the mucosa; ulcers; mucosal nodularity
Immunophenotyping	CD3+	CD3+ CD4+ (frequent) or CD8+ or CD4-/CD8- (rare)
Clonality pattern	Clonal or oligoclonal TCRγ rearrangement	Clonal or oligoclonal TCRγ rearrangement
Main differential diagnosis	Inflammatory bowel disease	Refractory coeliac disease, autoimmune enteropathy
Outcome	Indolent evolution	Indolent evolution
	Median survival time of 2 years	Persistent disease at a median follow up of 5 years
Treatment	No gold standard	No gold standard
	Chlorambucil and steroids most common	“Watch and wait” strategy, immunosuppressive agents, chemotherapy (CHOP regimen), anti CD52 monoclonal antibody

As previously mentioned, the pathogenesis of human indolent digestive T-cell LPD is poorly understood and the rarity of the disease in humans is the major factor limiting the identification of putative oncogenic events. In contrast, the fact that feline LGAL is more common suggests that it might be a suitable animal model for the human condition, to elucidate some of the pathogenic mechanisms underlying human indolent T-cell LPD of the gastrointestinal tract. Through domestication, humans, dogs and cats share a common environment and therefore display common signatures of coevolution, including epigenetic markers [166, 167]. This might explain why humans and pets are affected by closely-related diseases resulting from variations in genes expression altering conserved functional pathways [168]. The depth and quality of medical phenotyping in pets, associated with their facilitating haplotype structure and the development of genomics tools make them the most valuable mammalian models in medical genetics, including comparative oncology [169]. In the last two decades, dozens of disease-causing variants have been successfully identified in pets, most of them directly informative for humans [168]. Therefore, there is great potential for future research into feline LGAL not only to benefit cats, but also humans.

## Conclusion

Feline low-grade alimentary lymphoma (LGAL) is the most frequent intestinal neoplasm and is characterised by diffuse infiltration of monomorphic neoplastic T-cells in the gastro-intestinal tract. However, diagnosis is still a major challenge, mainly the difficulty in differentiate the condition from inflammatory bowel disease. That said, developments in immunohistochemical analysis and clonality testing have improved the confidence of clinicians in obtaining a correct diagnosis. The pathophysiology of feline LGAL still needs to be elucidated, not least the putative roles of infectious agents, environmental factors as well as genetic events. The most common therapeutic strategy is combination treatment with prednisolone and chlorambucil, and prolonged remission can often be achieved. However, different variations of this therapeutic strategy exist, and standardisation is needed.

Importantly, feline LGAL could be considered to be a potential animal model for human indolent digestive T-cell lymphoproliferative disease. By exploring the pathogenetic mechanisms involved in feline LGAL, it should be possible to improve understanding of the human disease, identify diagnostic and prognostic markers and develop effective therapeutic regimens. More broadly, such an approach could provide insights into the host-pathogen interactions occurring in the gastrointestinal tract and fundamental processes involved in physiological gut immunology.

## Abbreviations

AL: Alimentary lymphoma
EATL: Enteropathy-associated T-cell lymphoma
EE: Eosinophilic enteritis
EIL: Epitheliotropic intestinal malignant lymphoma
FcaGHV1: *Felis catus* gammaherpesvirus 1
FCEAI: Feline chronic enteropathy activity index
FeLV: Feline leukaemia virus
FFPE: Formalin-fixed and paraffin-embedded
FIV: Feline immunodeficiency virus
HE: Haematoxylin and eosin
HGAL: High-grade alimentary lymphoma
Hhe: *Helicobacter heilmannii*
HIV: Human immunodeficiency virus
IBD: Inflammatory bowel disease
IHC: Immunohistochemistry
LGAL: Low-grade alimentary lymphoma
LGLL: Large granular lymphocytic lymphoma
LPD: Lymphoproliferative diseases
LPE: Lymphoplasmacytic enteritis
MALT: Mucosa-associated lymphoid tissue
MEITL: Monomorphic epitheliotropic intestinal T-cell lymphoma
NA: Not available
NHL: Non-hodgkin lymphomas
NK: Natural killer
PARR: PCR for antigen receptor rearrangements
PCR: Polymerase chain reaction
PO: Per Os
SC: Subcutaneous
SIV: Simian immunodeficiency virus
WHO: World Health Organisation
